# Chronic Myeloid Leukemia and Hepatoblastoma: Two Cancer Models to Link Metabolism to Stem Cells

**DOI:** 10.3389/fonc.2016.00095

**Published:** 2016-04-14

**Authors:** Maria Grazia Cipolleschi, Ilaria Marzi, Elisabetta Rovida, Persio Dello Sbarba

**Affiliations:** ^1^Department of Experimental and Clinical Biomedical Sciences “Mario Serio”, Università degli Studi di Firenze, Florence, Italy

**Keywords:** cancer stem cells, microenvironment, metabolism, hypoxia, glucose shortage, chronic myeloid leukemia

## Abstract

Low oxygen tension is a critical aspect of the stem cell niche where stem cells are long-term maintained. In “physiologically hypoxic” stem cell niches, low oxygen tension restrains the clonal expansion of stem cells without blocking their cycling, thereby contributing substantially to favor their self-renewal. The capacity of stem cells, hematopoietic stem cells in particular, to reside in low oxygen is likely due to their specific metabolic profile. A strong drive to the characterization of this profile emerges from the notion that cancer stem cells (CSC), like normal stem cells, most likely rely on metabolic cues for the balance between self-renewal/maintenance and clonal expansion/differentiation. Accordingly, CSC homing to low oxygen stem cell niches is the best candidate mechanism to sustain the so-called minimal residual disease. Thus, the metabolic profile of CSC impacts long-term cancer response to therapy. On that basis, strategies to target CSC are intensely sought as a means to eradicate neoplastic diseases. Our “metabolic” approach to this challenge was based on two different experimental models: (A) the Yoshida’s ascites hepatoma AH130 cells, a highly homogeneous cancer cell population expressing stem cell features, used to identify, in CSC adapted to oxygen and/or nutrient shortage, metabolic features of potential therapeutic interest; (B) chronic myeloid leukemia, used to evaluate the impact of oxygen and/or nutrient shortage on the expression of an oncogenetic protein, the loss of which determines the refractoriness of CSC to oncogene-targeting therapies.

## Introduction

Low oxygen tension is a critical aspect of the environment where stem cells reside. On the basis of *in vitro* data, we were the first to put forward, in 1993, the hypothesis that hematopoietic stem cell (HSC) niches, where HSC are physiologically hosted *in vivo*, are bone marrow areas maintained at relatively low oxygen tension (1). Our results, limited to short-term repopulating HSC, were later confirmed by others and extended to comprise long-term repopulating HSC (2). The capacity of HSC, but not of hematopoietic progenitor cells (HPC), to home in low oxygen tissue areas is crucial to ensure HSC self-renewal and long-term maintenance. This capacity is likely due to the specific metabolic profile of HSC. We also found that low oxygen does not inhibit HSC cycling but limits cycling to support HSC self-renewal (3). Thus, the environment of “physiologically hypoxic” niches (4) contributes substantially to maintain stem cell potential. A number of excellent reviews addressed the relationship of niche environment to oxygen tension and blood supply in bone marrow. Moreover, the overall architecture of niche, which includes stromal cells, extracellular matrix, and soluble or matrix-bound cytokines has also been extensively described (5, 6). The physiological role of relatively low oxygen tensions in the regulation of stem cell compartments was further supported by later studies on pluripotent embryonal stem cells. Indeed, in the developing embryo, the inner cell mass of blastocyst is a relatively “hypoxic” (pO_2_ < 2%) structure (7), where embryonal stem cells largely rely on glycolytic ATP generation independently of oxygen shortage (8), i.e., exploiting the “aerobic” glycolysis or Warburg effect (9).

Cancer cell populations are usually characterized by a marked phenotypical heterogeneity to include differentiating cells as well as cancer stem cells (CSC). CSC seem to rely on environmental cues similar to those characterizing physiological stem cell niches for either their survival or the regulation of balance between self-renewal/maintenance and clonal expansion/differentiation. CSC continuously interact with non-neoplastic components of the niche, which decisively contribute to the maintenance of CSC *via* their protection from insults of different nature coming from outside the niche. These insults range from the physiological pressure of cytokines boosting clonal expansion at the expense of stem cell maintenance to the action of therapeutic agents (10, 11). The location itself of the niche may represent a powerful protection factor. Indeed, being far away from blood vessels implies the exposure to significantly lower concentrations of systemically administered drugs than in the rest of tissue. Furthermore, although CSC are allowed to cycle in the niche, their slow cycling or even quiescence provides an obvious protection from the effects of chemotherapeutic agents designed to suppress proliferating cells (12). On the other hand, CSC hosting in niches at extremely low oxygen tension has the straightforward consequence that CSC are protected from the effects of reactive oxygen radicals (ROS), which are instead typically generated in better oxygenated tissue areas. This represents a serious problem in relation to the effectiveness of radiotherapy on CSC, which largely relies on ROS generation to induce DNA damage in tumor cells (13).

Moreover, it is worth noting here that CSC have intrinsic properties that make them resistant to treatments. These properties, including the expression of aldehyde dehydrogenase and of the two ABC transporters MDR1 and ABCG2, which drive drug efflux from cells, have been excellently reviewed elsewhere (14). The association between multidrug resistance (MDR) and stem cell markers in human chronic myeloid leukemia (CML) cell lines has been clearly shown (15). The combination of CSC-intrinsic properties with the CSC-sheltering effects of stem cell niche makes it impossible to obtain a homogeneous therapeutic efficacy on the whole cancer tissue. In this scenario, CSC homing within niches is the best candidate mechanism to sustain minimal residual disease (MRD). Such a clinical state may determine late relapse of disease even in patients who brilliantly responded to therapy undergoing to complete remission. Low oxygen turns out to represent “the,” or at least “one of the,” most important factors ensuring MRD persistence (16). This implies that CSC, like normal stem cells, are capable to metabolically adapt to low oxygen. More in general, CSC are capable to adopt a nutrient uptake pattern and energy metabolism profile that favors their homing into niches (i.e., an adequate interaction between the oxygen-sensing and the nutrient-sensing signaling pathways). To conclude, one can affirm that the metabolic profile of CSC heavily impacts cancer response to therapy.

Modern oncology is testing selective CSC targeting as a means to eradicate neoplastic disease (17), aiming at their cure rather than care. The latter outcome is, instead, typical of actions directed to suppress the proliferating bulk of cancer population. Our “metabolic” approach to this challenge was based on two different experimental models. We used AH130 ascites hepatoma cells (18) to try to identify some step of metabolic pathways, which is particularly vulnerable in CSC adapted to low oxygen. On the other hand, we choose CML as a model disease to evaluate the therapeutic targeting of CSC in cell populations where low oxygen determines the suppression of an oncogenetic protein and the consequent loss of oncogene addiction.

## AH130 Cells as a Prototype of Cancer Stem Cells

The metabolic studies we summarize here were necessarily preceded by a reappraisal of the nature of the AH130 cell population. This hepatoma can be maintained indefinitely *via* serial transplantations in the rat peritoneal cavity, where huge amounts of immature cells are produced each time. AH130 cells express fundamental embryonic transcription factors (ETF), such as Nanog, Klf4, and c-Myc. When peritoneal cavity is cell-saturated and cell growth is arrested, glucose concentration as well as oxygen tension approach 0. In spite of that all hepatoma cells are alive, 75% of which expressing Nanog and more than 90% the stem cell marker CD133 (19). Thus, the AH130 hepatoma is an inexhaustible source of stem cells, which provides a decisive advantage in view of the characterization of their metabolic profile.

**Figure 1:**
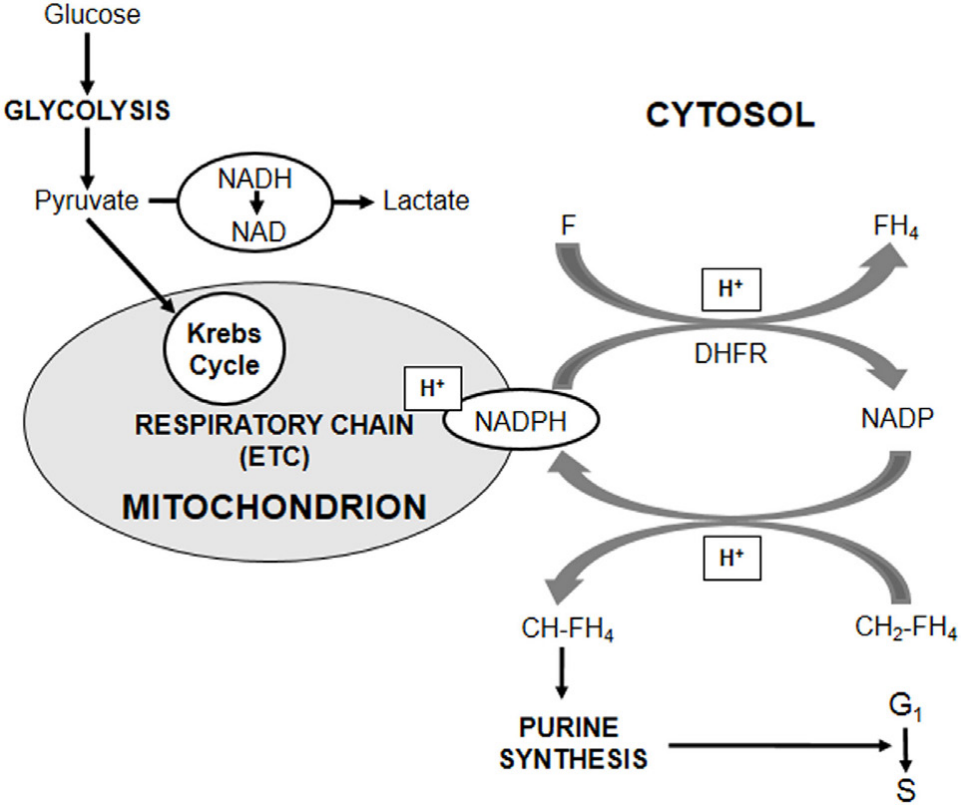
**Role of cellular Redox state in the control of cell cycling**. The core of the metabolic network controlling AH130 hepatoma cell cycling is the cellular RedOx state expressed by the cytosolic NADP/NADPH ratio. The transfer of reducing equivalents (H^+^) from methylene-tetrahydro-folate (CH_2_-FH_4_) to NADP, generating methenyl-tetrahydro-folate (CH-FH_4_) and NADPH, is a limiting step of the assembly of purine ring required for the amplification of purine pools indispensable for the G_1_–S transition of mitotic cycle. An accumulation of cytosolic NADPH inhibits cell recruitment into S. A fundamental role in the regulation of NADP/NADPH ratio is played by folate (F), whose reduction to tetrahydro-folate (FH_4_) by dehydrofolate-reductase (DHFR) generates NADP. When DHFR activity is impaired by the addition of its inhibitor Methotrexate or of an excess of the reaction product (FH_4_), NADPH increases with the consequent reductive shift of NADP/NADPH ratio and the inhibition of purine synthesis. However, the major antagonist of this shift is the transfer of cytosolic reducing equivalents onto the mitochondrial ETC through suitable shuttles, accounting for the crucial role of ETC in purine synthesis. This transfer is antagonized whenever ETC, although not inhibited, is saturated by reducing equivalents produced by oxidizable substrates of the Krebs cycle, such as pyruvate.

Well in keeping with the scenario summarized in the Introduction, AH130 cells are, like other stem cells, adapted to low oxygen (20). The metabolic profile of AH130 cells is indeed centered on an extremely high capacity of converting pyruvate into lactate, so that cells, when exposed *ex vivo* to high glucose concentrations (upto 15 mM) in air, convert up to 80% of the available glucose to lactate, exhibiting an excellent example of Warburg metabolism. This enormous energy waste implies that the elimination of pyruvate produced by aerobic glycolysis is a primary exigency, due to a detrimental effect of pyruvate accumulation on G_1_–S transition. A key finding to understand the mechanism of this effect was that the addition of exogenous pyruvate to AH130 cells (incubated in air in the presence of 15-mM glucose) faithfully mimicked that of antimycin A, which blocks electron transport chain (ETC) inhibiting its complex III. On the contrary, 2,4-dinitrophenol, which uncouples (but does not block) ETC function from ATP generation, did not mimic the effects of exogenous pyruvate or antimycin A. This indicates that in AH130 cells glycolytic energy production is sufficient and that the role of ETC is related instead to the fact that some pyruvate cannot be converted to lactate and needs to be oxidized. In other words, ETC seems necessary to prevent a detrimental accumulation of reducing equivalents coming from oxidizable substrates. In turn, an excess of oxidizable substrates can saturate ETC. In this respect, the differentiation state of cells is critical. ETC saturation is easily prevented in cells endowed with large mitochondrial equipment (“transit” progenitor cells characterized by high proliferation rate), but easily produced in cells with few mitochondria (21). AH130 cells possess, as a consequence of their adaptation to low oxygen, a very scanty mitochondrial apparatus, easily saturable by pyruvate at concentrations which are instead easily catabolized by progenitor cells. Thus, AH130 cells represent the prototype of stem cells that are vulnerable to physiological metabolites totally innocuous to non-stem cells. In conclusion, AH130 cell recruitment into S can be limited in two ways: (a) hindering respiration, either under oxygen shortage or by impairing the electron transport to oxygen using ETC inhibitors (antimycin A) and (b) saturating the ETC with an excess of oxidizable substrates (pyruvate). The metabolic network vulnerable to pyruvate is outlined in Figure 1. AH130 cell recruitment into S implies, to control the neo-synthesis of DNA, a tight complementation of glycolysis, cellular RedOx state, and folate metabolism. This recruitment is controlled by a cytosolic NADP-dependent step of folate utilization in the synthesis of purine ring, a step where NADPH produced is re-oxidated through the transport of reducing equivalents (electrons) to ETC (21, 22).

## Chronic Myeloid Leukemia and the Metabolic Control of Oncogene Addiction

Chronic myeloid leukemia is determined by a reciprocal translocation between chromosomes 9 and 22, resulting in the formation of the chimeric BCR/Abl protein (hereafter “BCR/Abl”) that functions as a constitutively active tyrosine kinase. Tyrosine kinase inhibitors (TKI), such as imatinib-mesylate (IM), are highly effective to suppress BCR/Abl enzymatic activity and to treat chronic phase CML patients. However, in a large majority of patients, IM does not efficiently kill leukemic stem cells (LSC), the crucial event to cure CML (23). It is becoming clear that second-/third-generation TKI remain unable to eradicate LSC. Therefore, strategies directed to hit TKI-resistant LSC aiming at targets different from BCR/Abl are intensely sought after.

Mechanisms traditionally believed to determine resistance to IM and TKI are (24): (a) mutations of *BCR*/*abl* gene within the tyrosine kinase domain (primary or secondary to treatment), (b) amplification of *BCR*/*abl*, (c) mutations outside *BCR*/*abl* determining BCR/Abl-independent survival and proliferation, (d) enhanced activity of drug exporters, and (e) quiescence. A novel mechanism of resistance to IM emerged from our studies *in vitro*. We showed indeed, using CML cell lines, that a very low oxygen tension (0.1% oxygen) in the incubation atmosphere maintains the stem cell potential while cell growth is inhibited and the oncogenic BCR/Abl protein is suppressed (25, 26). Being deprived of BCR/Abl, LSC selected in low oxygen are independent of BCR/Abl signaling, i.e., they lack oncogene addiction (27). In spite of this, LSC selected in low oxygen remain genetically leukemic, so that they are capable to regenerate a BCR/Abl-expressing/dependent progeny (25, 26). Thus, BCR/Abl suppression in low oxygen is not a genetically blocked event, but a fully reversible phenotypical adaptation. This fact is in keeping with the “*chiaroscuro* stem cell” model proposed to describe the relationship between the HSC and HPC phenotypes (28). The refractoriness to TKI of LSC of CML adapted to low oxygen is a straightforward consequence of the transient and reversible suppression of their molecular target. We defined “environment-enforced BCR/Abl suppression” this mechanism of resistance to TKI. This mechanism does not require, to explain the onset of resistance, to postulate the occurrence of permanent changes in a CML subclone due to secondary mutations (29). Our *in vitro* findings are strongly supported by the observation that LSC do not depend on BCR/Abl kinase activity for their survival (30), as well as by the clinical evidences that MRD and the related CML relapse following successful IM treatment is usually sustained by cells expressing wild-type BCR/Abl (31). On the other hand, our model well explains: (a) the discrepancy observed in CML patients between the expression of *BCR*/*abl* transcript and that of BCR/Abl protein (32) and (b) the IM resistance of CML progenitors shown to be *BCR*/*abl*-positive by FISH or PCR (33), which we believe to be in fact transcript-positive/protein-negative cells.

The environment-enforced BCR/Abl-negative/TKI-resistant phenotype implies that LSC are metabolically adapted to home within the “hypoxic” stem cell niches of bone marrow where HSC physiologically reside (see Introduction). Interestingly, we found that LSC adaptation to low oxygen and BCR/Abl suppression are not necessarily linked to quiescence (unpublished data). This is in keeping with the findings we obtained for HSC indicating that low oxygen restrains and redirects their cycling to support self-renewal (3). The capacity of cycling in low oxygen is obviously crucial to allow LSC self-renewal within the stem cell niche and, therefore, to maintain MRD. On the other hand, as cell cycling is necessary for the permanent incorporation of mutations in a cell population, this capacity appears the key factor for neoplastic progression during silent/subclinical phases of the disease. Thus, the metabolic adaptation of LSC seems to warrant all the features necessary to keep CML going in a therapy-resistant fashion.

**Figure 2:**
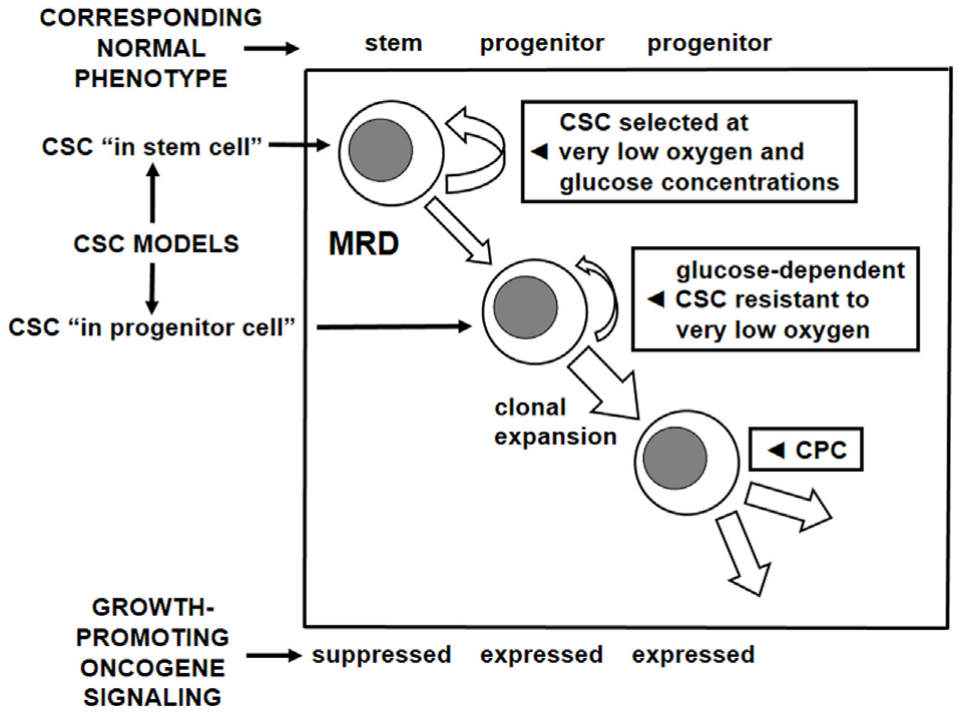
**Cancer stem cell models, oncogene dependence, and metabolic profile**. Correspondence to the normal stem and progenitor cell phenotypes of two complementary subsets of CSC identified on the basis of two models for their generation. Relationship of these subsets to oxygen and glucose supply in tissue microenvironment as well as to the activity of growth-promoting oncogene signaling. CSC, cancer stem cell; CPC, cancer progenitor cell; MRD, minimal residual disease; the width of arrows corresponds to the hypothesized level of activity.

A first attempt to characterize the adaptation of LSC to a low oxygen environment and its relationship to BCR/Abl suppression showed that this suppression occurs when, in low oxygen, glucose approaches exhaustion (26). Therefore, a low-oxygen/low-glucose environment appeared as the appropriate condition for the maintenance of TKI-refractory CSC sustaining MRD (CSC/MRD). In this respect, it is worth pointing out that glucose exhaustion, under our standard experimental conditions, is reached only after 7 days of incubation in low oxygen. Thus, it is evident that a cell subset exists which can stand low oxygen but still takes advantage of glucose availability and BCR/Abl signaling. It is likely that this cell subset includes CSC that dedicate most of their proliferative potential to oncogene-driven clonal expansion, although they maintain a – probably low – level of self-renewal. We are convinced that this CSC subset and the CSC/MRD subset correspond to the two CSC models originally proposed as alternative: the “CSC in normal stem cell” and the “CSC in normal progenitor cell” (34). Our CML data strongly suggest considering rather these models as complementary. The relationships among CSC models, self-renewal/clonal expansion balance, MRD, role of growth-promoting oncogene signaling, and dependence on oxygen and/or glucose supply are summarized in Figure 2.

## Concluding Remarks

A question arising from the information summarized above was whether the Yoshida’s hepatoma and the CML models could complement each other contributing to define a unifying scenario for CSC adaptation to low oxygen. We believe that one can answer this question affirmatively. Indeed, AH130 hepatoma cells exhibit a Warburg-type metabolic profile *in vitro* and *in vivo* until oxygen is available, adapt to oxygen shortage relying on glycolysis, and undergo mitotic arrest when glucose shortage complicates oxygen shortage. Thus, the AH130 hepatoma represents a phenotypically homogeneous CSC-like cell population, which is very well suitable for biochemical, molecular, as well as biological studies. CML cell lines, on the other side, are heterogeneous populations, which retain the capacity to differentiate under different environmental conditions, yet, including a cell subset with stem cell traits. However, CML, like hepatoma, cells exhibit the aerobic or the anaerobic glycolytic profile depending on oxygen availability. Moreover, depending on glucose availability or shortage, LSC of CML cells are subjected, rather than to growth promotion/arrest, to a switch between two different CSC phenotypes characterized by different metabolic profiles. This phenotypical difference reflects the expression or suppression of the oncogenetic protein responsible for the disease and underscores the partition of CSC compartment in two subsets, which are dynamically related to each other and reversibly linked to environmental conditions. These subsets drive either the florid state of disease (CSC) or the maintenance of disease during clinical remission (CSC/MRD). Interestingly, the reversibility of the two CSC phenotypes emerged from studies carried out using stabilized CML cell lines, in particular, K562 cells. This strengthens considerably the issue of the genetic identity of the two CSC subsets. A high percentage of K562 cells expresses ETF and exhibits a high level of sensitivity to pyruvate addition. Thus, these cells appear to share with AH130 cells important aspects of gene expression and metabolism profiles. On the other hand, it is also of high interest that the most relevant results obtained using stabilized CML cell lines were confirmed using a number of different primary explants from CML patients (26, 35). These findings allow to export the above conclusions about the CML stem cell compartment to clinical settings.

Stem cells, besides being characterized by a high glycolytic activity, also consume oxygen *via* a functional ETC (36). Referring to CML, the emerging question is whether, when glucose is exhausted, this oxidative activity can be sufficient to sustain energy production from other substrates (29). This scenario may describe appropriately the metabolic profile of CSC/MRD, whereas CSC and cancer progenitor cells (CPC) would follow the AH130-type glucose-dependent metabolic pattern. Metabolic differences between quiescent or slow-cycling CSC/MRD and rapidly proliferating CSC/CPC are emerging from recent literature (37, 38). On the other hand, it has been demonstrated that a dormant subset of pancreatic cancer cells capable to survive oncogene ablation (like CML cells subjected to environment-enforced BCR/Abl suppression) is responsible for tumor relapse, has CSC features, and relies on oxidative phosphorylation for survival (39). CML studies led us to envision a two-tier model of CML stem cell niche where different LSC subsets establish a sort of “metabolic symbiosis” conceptually similar to that shown between cancer cells and stroma within solid tumors (29). According to this model, a drop of oxygen tension in the niche periphery would stimulate glycolysis therein *via* the activation of transcriptional activity mediated by hypoxia-inducible factor 1α (HIF1α). The consequent high rate of glucose consumption would determine a sharp decrease of pH and increase of lactate concentration in the niche core. There, low pH would inhibit HIF1α, and available substrates, such as lactate itself, would be oxidized to produce energy suitable for LSC survival.

In summary, the combination of data from the hepatoma and CML models led us to hypothesize the following coupling between metabolic profiles and functional subsets of cancer cell populations:

(A) Low oxygen-sensitive CPC (proliferation directed to clonal expansion coupled with differentiation); high-level oncogenetic proliferative signaling (e.g., BCR/Abl expressed); high-level oxidative energy production; and many (and elongated/*cristae*-rich) mitochondria.
(B) Low oxygen-resistant self-renewing CSC (proliferation coupled with commitment to clonal expansion); reduced but not suppressed oncogenetic proliferative signaling (e.g., BCR/Abl undergoing suppression); upregulated glycolytic energy production, sustained by glucose availability (maintained even if oxygen supply is restored – Warburg effect); few (and rounded/*cristae*-poor) mitochondria; and low-level oxidative activity directed, rather than to provide energy, to prevent a detrimental accumulation of reducing equivalents produced in high quantities by upregulated glycolysis.
(C) Low oxygen-adapted self-renewing CSC (proliferation uncoupled with commitment to clonal expansion); suppressed oncogenetic proliferative signaling (e.g., BCR/Abl suppressed); downregulated glycolytic energy production due to glucose exhaustion; few mitochondria; and low-level oxidative activity sufficient to sustain energetically the slow-cycling/quiescent cell subset responsible for MRD.

On the basis of all above, the use of metabolic inhibitors to target CSC emerges as a therapeutic strategy well worth being explored if one aims at the cure of disease, i.e., at its eradication. In this respect, a number of attempts have been actually carried out using inhibitors of the best known pathways of energy metabolism, such as glycolysis and mitochondrial function. These inhibitors include the glucose analog 2-deoxyglucose, which accumulates in cells and inhibits hexokinase; dichloroacetate, an inhibitor of pyruvate dehydrogenase kinase, which forces pyruvate to mitochondrial metabolism; metformin, an anti-diabetic drug endowed with insulin-dependent and direct insulin-independent anticancer effects (40–43).

Perhaps even more interesting therapeutic alternatives are represented by the use of physiological substrates related to “energy” metabolism. We have previously shown that peculiar metabolic features of cell adaptation to, and survival in, low oxygen imply growth restriction points that can be targeted by physiological factors, such as pyruvate, tetrahydrofolate, and glutamine (20). For instance, in the presence of pyruvate (upto 20 mM), tumors of various histogenesis (melanoma and neuroblastoma, in addition to AH130 hepatoma and CML) undergo growth inhibition *in vitro*, to levels apparently proportional to their degree of anaplasia, being this inhibition maximal (up to 90%) for AH130 cells. Remarkably, pyruvate is innocuous, even at the highest doses, to normal differentiated cells. Thus, cancer growth can be attacked not only *via* the targeting of metabolic pathways in general but also *via* their targeting, in particular, using physiological substrates. The possibility of transferring the latter strategy to preclinical settings is being addressed in our laboratory.

## Author Contributions

MGC, IM, and ER carried out personally or supervised the collection of data mentioned in this review. MC and PDS wrote the manuscript. IM and ER made the figures and edited the manuscript.

## Conflict of Interest Statement

The authors declare that the research was conducted in the absence of any commercial or financial relationships that could be construed as a potential conflict of interest.
